# Impact of molecular imaging in preclinical cancer research

**DOI:** 10.1186/1753-6561-7-S2-K4

**Published:** 2013-04-04

**Authors:** Carola Heneweer, Holger Kalthoff

**Affiliations:** 1 Department of Radiology; UKSH-Campus Kiel, Arnold-Heller-Str. 3, 24105 Kiel, Germany; 2 Institute for Experimental Cancer Research, Comprehensive Cancer Center North; UKSH-Campus Kiel, Arnold-Heller-Str. 3, 24105 Kiel, Germany

## 

Development and progression of any cancer disease are the result of various alterations at the cellular and molecular level. This comprises changes in expression of genes, display of surface molecules, composition of extracellular matrix, and homing of circulating cells to the tumor site. Most of these changes occur long before morphological changes can be detected by conventional methods.

Molecular imaging is aimed at the non-invasive in vivo characterization and measurement of these processes to assess therapy effects more prompt than classic morphological and functional imaging can provide. Additionally, visualization of these processes would provide more precise information about the disease expansion. Beyond that, novel therapy regimens such as immunotherapies require methods for tracking the therapeutic cells. Different imaging modalities are used for these purposes, originally established in cell biology labs like fluorescence imaging (FLI), bioluminescence imaging (BLI), and photoacoustic imaging (PAI) as well as in clinical routine like magnetic resonance imaging (MRI), computed tomography (CT), positron emission tomography (PET), single photon emission CT (SPECT), and ultrasound (US).

Variations in endogenous tissue contrast can be utilized for certain applications, e. g. alterations in oxygen saturation lead to signal changes in PAI and dedicated MRI sequences like BOLD sequences. However, specific contrast agents need to be designed in most assignments. These molecules usually consist of a targeting moiety that binds to molecules of interest, indicates certain functional states of the tissue, or is modified by specific enzymes on the one hand. On the other hand, another moiety is needed that changes tissue contrast in order to be detected by the chosen modality, like fluorophores for FLI, microbubbles for US, e.g. gadolinium for MRI, e.g. ^18^F for PET and e.g. ^99m^Tc for SPECT. These contrast agents can either be injected into the animals whereby pharmacokinetics of the molecule itself determines the imaging protocol. Alternatively, they can be used for cell labelling in order to track these cells after injection into the animals. Most of these techniques in general provide the opportunity to be translated into clinical routine.

This toolbox of endogenous and exogenous contrast agents is completed by reporter gene imaging that allows detecting changes in gene expression. This technique is in most cases limited to preclinical imaging. To visualize changes of gene expression, genetic sequences are used that code for different fluorescent proteins for FLI, different luciferases for BLI, herpes simplex virus tyrosine kinase (hsv-tk) for PET or e.g. iron-binding or iron-storage proteins for MRI. These gene sequences are brought under the control of the promoter of interest. The vectors constructed this way are then either transfected into tumor cells for preclinical transplantation models of cancer or used for the generation of transgenic animals.

Pancreatic ductal adenocarcinoma (PDAC) represents one of the malignancies with the poorest prognosis where incidence equals mortality. Despite considerable advances in the understanding of the molecular mechanisms involved in the carcinogenesis of PDAC during the last decade the survival of the disease was not significantly improved over the last 40 years. Therefore, novel therapeutic approaches are urgently needed. Preclinical animal imaging can provide accurate measures of tumor progression non-invasively. Molecular imaging can in addition help, a) to detect cellular and molecular processes in vivo avoiding artefacts caused by tissue collection and preservation, b) to visualize specific therapy effects on certain signalling pathways over time, and c) to reduce animal numbers needed due to its non-invasiveness.

PDAC is characterized by early spreading of metastatic cells and a high rate of local and distant recurrent disease even after complete surgical removal of the primary lesion as defined by histology. One key factor for this aggressiveness seems to be due to the high susceptibility towards inflammatory signals, part of which are acting in an autocrine manner since PDAC cells have been found to frequently express simultaneously ligands and corresponding receptors. From a surgical point of view local recurrent disease as well as distant metastases (mostly in the liver) limit the success of this curative therapeutic attempt, for which less then 20% of patients are eligible. There is strong evidence that inflammation drives these sometimes rapid and fulminant recurrences. Adjuvant chemotherapy has been shown to improve the outcome yet no long-term survival is achieved. Numerous different contrast agents for all modalities are available to detect surrogate markers of inflammation, such as endothelial adhesion molecules like E-selectin or VCAM, as well as enzymatic activity e.g. of matrix metalloproteases, caspases or cathepsins. Additionally, invasion of immune cells can be monitored by reporter gene imaging.

A plethora of molecular alterations are thought to be responsible for the profound chemoresistance. Besides classical hallmarks of cancer such as mutations in the K-ras oncogene and the p53 tumor suppressor, the constitutive or inducible activity of transcription factor pathways is characteristic for PDAC. The nuclear factor-κB (NF-κB) has been shown besides others to be crucial for tumor development and apoptosis resistance mechanisms in this context. We have shown that NF-κB contributes to non-apoptotic signalling of death receptors, “normally” inducing apoptosis upon death-ligand-driven activation. Thus, targeting this pathway by established recombinant inhibitors or new drugs including natural compounds with anti-inflammatory potential might have great potential for future therapeutic concepts.

In summary, in vivo animal models are a key element of understanding the processes of tumor development and progression as well as of validating novel strategies for tumor therapies. Clinically adapted animal models allow distinguishing between adjuvant, palliative and neo-adjuvant concepts. Preclinical animal imaging and especially molecular imaging can help to visualize molecular and cellular processes during the course of the disease non-invasively in vivo, and will allow a more valid validation of any novel therapeutic regime.

## Competing interests

There are no competing interests in this presentation.

